# Analysis methods for covariate-constrained cluster randomized trials with time-to-event outcomes

**DOI:** 10.1186/s12874-025-02465-w

**Published:** 2025-01-22

**Authors:** Amy M. Crisp, M. Elizabeth Halloran, Matt D. T. Hitchings, Ira M. Longini, Natalie E. Dean

**Affiliations:** 1 Department of Biostatistics, University of Florida, Gainesville, Florida USA; 2 Department of Biostatistics, University of Washington, Seattle, Washington USA; 3 Vaccine and Infectious Diseases Division, Fred Hutchinson Cancer Center, Seattle, Washington USA; 4 Department of Biostatistics and Bioinformatics, Emory University, Atlanta, Georgia USA

**Keywords:** Clinical trial design, Cluster-randomized, Constrained randomization, Time-to-event, Permutation test

## Abstract

**Background:**

Cluster randomized trials, which often enroll a small number of clusters, can benefit from constrained randomization, selecting a final randomization scheme from a set of known, balanced randomizations. Previous literature has addressed the suitability of adjusting the analysis for the covariates that were balanced in the design phase when the outcome is continuous or binary. Here we extended this work to time-to-event outcomes by comparing two model-based tests and a newly derived permutation test. A current cluster randomized trial of vector control for the prevention of mosquito-borne disease in children in Mexico is used as a motivating example.

**Methods:**

We assessed type I error rates and power between simple randomization and constrained randomization using both prognostic and non-prognostic covariates via a simulation study. We compared the performance of a semi-parametric Cox proportional hazards model with robust variance, a mixed effects Cox model, and a permutation test utilizing deviance residuals.

**Results:**

The permutation test generally maintained nominal type I error—with the exception of the unadjusted analysis for constrained randomization—and also provided power comparable to the two Cox model-based tests. The model-based tests had inflated type I error when there were very few clusters per trial arm. All three methods performed well when there were 25 clusters per trial arm, as in the case of the motivating example.

**Conclusion:**

For time-to-event outcomes, covariate-constrained randomization was shown to improve power relative to simple randomization. The permutation test developed here was more robust to inflation of type I error compared to model-based tests. Gaining power by adjusting for covariates in the analysis phase was largely dependent on the number of clusters per trial arm.

**Supplementary Information:**

The online version contains supplementary material available at 10.1186/s12874-025-02465-w.

## Background

The cluster randomized trial, in which groups or units (e.g., schools, hospitals, census tracts) are randomized into treatment arms and observations are taken from individuals within those units, provides an alternative to individually randomized trials [1]. They are particularly useful when the treatment is to be implemented at the group level, or when individual randomization is not logistically feasible. However, cluster randomized trials are often performed using a small number of clusters ($\le$ 20) [2], leading to the prospect of chance imbalance between the treatment arms for both individual-level and cluster-level covariates. Such an imbalance can be especially concerning for those covariates that are known or considered to be prognostic of the primary endpoint [3]. Not only does an imbalance potentially pull type I error away from the nominal value [4] and raise questions about the validity of any asymptotic assumptions required for causal inference, but obtaining covariate balance across treatment arms also increases the power of a trial by reducing variability [5]. Furthermore, studies that attempt to compensate for any imbalances during the analysis phase—rather than the trial design phase—may find that the unadjusted and adjusted results differ from each other, sometimes even providing different conclusions [5]. This may raise questions about bias and the transparency of the trial as well as lead to confusion about the interpretation.

Several methods exist for dealing with these imbalances during the design phase of cluster randomized trials, including stratification, matching, minimization, and covariate-constrained randomization [6]. The most recently developed of these is covariate-constrained randomization, which is achieved by selecting a final allocation from a set of randomization schemes that balances certain covariates within a given tolerance [4]. An example of such a tolerance would be ensuring that the average annual income for clusters in the treatment arm is within $\pm 10\%$ of that in the control arm. An alternative method for achieving balance is to select the final allocation from those schemes in the lowest *q* percentile of a calculated “imbalance score” [3]—often a weighted squared difference of covariate means between arms. Once a final list of balanced allocations is compiled and checked for statistical validity (i.e., ensuring that each cluster is randomized independently of all other clusters [4]), one of them is randomly selected for use. Finally, one arm is randomly selected to be the treatment arm.

While the use of covariate-constrained randomization is growing, research is limited on the performance of methods used for analyzing the data from these studies. Li et al. published two simulation studies that explored analyses from cluster randomized trial data using constrained randomizations compared to simple randomizations. The first simulation study focused on type I error and power for continuous outcomes [7] while the second extended the results to binary outcomes [8]. They compared unadjusted analyses to analyses that adjusted for the covariates used in the covariate-constrained randomization. Furthermore, they considered both prognostic (i.e., covariates that are predictive of the outcome of interest) and non-prognostic covariates for the constrained randomization procedure in the case of the binary outcomes. These simulation studies found, in part, that permutation-based tests were best at maintaining nominal type I error across the intraclass correlation coefficient (ICC) and sample sizes. In the case of the permutation tests for constrained randomization, it was found that it is important to properly specify the permutational distribution by limiting it to the constrained randomization space rather than including all possible allocations. They also highlighted the importance of the covariates being prognostic, as this is where a gain in power can be achieved by adjusting for covariates.

We conducted simulations extending these models within a survival analysis framework. Time-to-event outcomes are frequently used in cluster randomized trials in a variety of specific contexts (e.g., participants may have different periods of surveillance or there may be loss to follow-up). The aims of this extension were (i) to compare simple randomization with constrained randomization using either prognostic or non-prognostic covariates, and (ii) to compare covariate-adjusted and unadjusted analyses. For the covariate adjustment, we considered three approaches: a semi-parametric Cox proportional hazards model using a mixed effects (frailty) term, a Cox proportional hazards model using a robust variance, and a permutation test using deviance residuals which we have derived for this purpose. The resulting power and type I error from the three statistical tests were examined.

In the Methods section, the simulations for the time-to-event data are explained in detail along with the derivation of the permutation test, and the results are presented in the Results section. In the Motivating example section, we then provide a motivating example from a cluster-randomized trial of vector control for the prevention of mosquito-borne illness in Mexico. Finally, the conclusions and limitations of this simulation study are presented in the Discussion section.

## Methods

### Data generation

Consider a cluster randomized trial with two arms containing *g* clusters in each arm and *n* participants in each cluster, for a total sample size of $2\times g\times n$. We generated time-to-event data under an exponential proportional hazards model with the following rate parameter:

1 $$\begin{aligned} \lambda _{ij} = \lambda _0 \gamma _i e^{\varvec{x}^{\varvec{\prime }}_{\varvec{i}} \varvec{\beta } + T_i log\delta }, \end{aligned}$$

where $i=1,...,2g$ is the cluster index, and $j=1,...,n$ is the subject within each cluster. The treatment indicator $T_i$ was taken to be 0 for clusters in the control arm and 1 for clusters in the treatment arm. Four binary cluster-level covariates were independently generated to create $\varvec{x}_i$ using Bernoulli distributions, each with a 30% probability of being one. The baseline hazard, $\lambda _0$, was set to provide an expected cumulative incidence of 25% over the two-year study period for the average individual with all cluster-level covariates $\varvec{x}_i =0$ and $T_i=0$. The group-level frailty term for the $i^{th}$ cluster is $\gamma _i$. The hazard ratio $\delta$ is defined as $\lambda _1 / \lambda _0$, where $\lambda _1$ is the hazard rate in the treatment arm. For studying type I error, the hazard ratio $\delta$ was set to 1, and for studying power, $\delta$ was set to 0.50, providing a treatment efficacy (i.e., 1 - hazard ratio) of 50%. Administrative censoring was implemented at two years for all data sets.

To compare prognostic and non-prognostic covariates, the simulations were performed using multiple values of the coefficient vector $\varvec{\beta }$. Initial sensitivity simulations demonstrated that the value of $\beta >0$ had a large influence on the intraclass correlation coefficient (ICC) for the generated data, as measured by

2 $$\begin{aligned} \text {ICC} = \frac{\sigma ^2_b}{\sigma ^2_b + \sigma ^2_w}, \end{aligned}$$

where for $\varvec{\beta }=\varvec{0}$, $\sigma ^2_b$ is the variance of the uncensored mean outcomes between clusters, and $\sigma ^2_w$ is the measured variance of the uncensored outcomes within clusters, averaged across clusters. For any $\beta >0$, we used the estimation of ICC from generalized linear mixed-effects models with Gamma distributions as given by Nakagawa et al. [9]. While there is discussion in the literature about the lack of an ICC definition specifically for survival data, there is an established need to account for the correlation in clustered time-to-event data—particularly in the design phase [10]. One of the known problems with broader definitions like Eq. (2) is that it does not account for censoring [11], thus we measured the ICC of the generated data prior to applying administrative censoring. While Eq. (2) did not calculate an exact measure of ICC for this data, it provided a measure of correlation that is not attributable to the covariates.

A stronger relationship between the cluster-level covariates and the outcome (i.e., $\varvec{\beta }$ farther from 0) yielded higher between-cluster variance and therefore a higher ICC. This led to a situation in which any additional frailty term induced an extreme level of ICC above and beyond the random effect, making interpretation of comparisons impossible. With all elements of the vector $\varvec{\beta }$ being equal and fixed to one of the values from the set $\{0.25, 0.35, 0.50\}$ and no additional frailty (i.e., $\gamma _i=1$), the resulting data had ICC $=\{0.05, 0.08, 0.14\}$, respectively. To achieve a non-prognostic setting ($\varvec{\beta } = \varvec{0}$) with comparable ICC, we adjusted the group-level frailty term ($\gamma _i \sim \text {Gamma(shape} = 1/\sigma ^2 \text {, scale} = \sigma ^2$)) to generate variability between the clusters that is unexplained by the covariates. The values of $\sigma ^2$ that provided the previously stated ICC values were $=\{0.087, 0.143, 0.268\}$, respectively.

The simulations were run at two levels of *g*: 8 and 13 clusters per arm, with each cluster containing $n=100$ subjects for all simulations. For each combination of the parameters $\delta$, *g*, $\varvec{\beta }$, and ICC, $n_{sim}=$ 1,000 complete data sets were generated, and $n_{sim}=1$ was initialized from the same seed for all cases to allow for reproducibility.

For each combination of parameters, the simulated data sets were then cluster-randomized into two arms using either a simple randomization or constrained randomization procedure. The same cluster-level covariate matrix $\varvec{x}_{(4 \times 2g)}$ was used for both the simple randomization and the constrained randomization for each iteration of $n_{sim}$. However, the constrained randomizations were limited to those with the least amount of covariate imbalance between the two arms. Using the covariate matrix in each simulation, 20,000 different cluster randomization schemes were generated, and the imbalance score **B** was calculated for each scheme using Eq. (3) [3].

3 $$\begin{aligned} \varvec{B} = \sum \limits _{k=1}^4 \omega _k (\overline{x}_{Ak} - \overline{x}_{Bk})^2, \end{aligned}$$

where $\omega _k$ is the inverse of the variance of the $k^{th}$ cluster-level covariate across all clusters, the indices *A* and *B* refer to the two arms, and $\overline{x}_{Ak}$ is the average of the $k^{th}$ cluster-level covariate in arm *A* (and similarly for $\overline{x}_{Bk}$). This imbalance score was used to constrain the randomizations by keeping only those allocation patterns with scores in the bottom *q* percentile (after duplicate randomizations were removed). Of note, simple randomization is equivalent to setting $q=1$ and retaining all patterns. We considered values of $q=\{0.10, 0.01\}$, representing a constrained randomization and a highly constrained randomization, respectively.

### Analysis methods

The data generated as described in the previous section were analyzed using three different methods. Each simulation output was fit with both a Cox proportional hazards model with a robust variance term (using the coxph function in the survival package [12] in R [13] which uses a Huber sandwich estimator for the robust variance) and a mixed effects Cox proportional hazards model with a random effects term (using the coxme package [14] in R which assumes a Gaussian distribution for the random effect). Both of these analysis methods were performed with no covariate adjustment and with adjustments for the same covariates that were used for balancing the randomizations in the design phase. These analyses are denoted by the total number of covariates included in the model: $S=0,1,2,3,4$. Since all four covariates are independently identically distributed, they can be adjusted for individually without loss of generality.

In addition to the semiparametric model-based analyses above, a permutation test was performed for comparison. Several nonparametric tests have been developed over the years for survival data, and many of these have been extended to include permutation tests [15]. However, it is difficult to find permutation tests that allow for both censoring of the survival data and adjustment of multiple covariates in the analysis. For a binary outcome, Li et al. [8] utilized a permutation test based on the residuals from a logistic regression, as proposed by Gail et al. [16]. The permutation test statistic [8] is defined as

4 $$\begin{aligned} S_{\text{ residual } }= & g^{-1}\left( \sum \limits _{i=1}^{2 g} T_{i} \bar{r}_{i}-\sum \limits _{i=1}^{2 g}\left( 1-T_{i}\right) \bar{r}_{i}\right) \nonumber \\= & g^{-1} \sum \limits _{i=1}^{2 g}\left( 2 T_{i}-1\right) \bar{r}_{i}, \end{aligned}$$

where $\bar{r}_{i} = n_i^{-1} \sum \nolimits _{j=1}^{n_i} r_{ij}$ is the residual mean in cluster *i*. Under the null hypothesis, the residual means are exchangeable, and the permutation distribution can be found by simply permuting the values for $T_i$. We constrained the permutation space to those randomizations that were found to be sufficiently balanced based on the value of *q* [3].

To properly account for the right-censoring of the survival data, we applied the same test statistic but using deviance residuals in Eq. (4) [17]. In their paper, Gail et al. [16] state that these methods may be extended to other forms of regression, and show with simulations that the regression model need not be correctly specified to maintain nominal size, so long as the design is balanced at the group level. The simulated data were fit with a Cox proportional hazards model under the null hypothesis, i.e., assuming $\delta =1$, and adjusted for covariates ranging from $S=0,...,4$. A cluster term was included in the models for robust variance. The outputs from those models were then used to calculate the deviance residuals with the residuals function in the survival package in R, which employs the following definition:

5 $$\begin{aligned} r_{Dj} = \Biggl \{ sign(\delta _{j}-r_{Cj}) \sqrt{-2[\delta _{j}-r_{Cj} + \delta _j log(r_{Cj})]} \Biggr \}, \end{aligned}$$

where $r_{Dj}$ is the deviance residual for the $j^{th}$ individual, $\delta _j$ is 0 if the $j^{th}$ individual is censored and 1 otherwise, and $r_{Cj}$ is the Cox-Snell residual for proportional hazards models $r_{Cj} = exp(\varvec{x}^T_j \hat{\varvec{\beta }}) \hat{H}_{0}(y_j)$. Here, $\hat{H}_{0}(y_j)$ is the baseline hazard function evaluated at $y_j$, the observed outcome for the $j^{th}$ individual. Monte Carlo standard errors (MCSE) were calculated for each analysis result following guidance for simulation studies [18]. These standard errors are depicted in the figures as error bars and are presented in parentheses in the tables.

## Results

The type I error and power results are presented in terms of the percent of $n_{sim}=1000$ simulations for which the null hypothesis ($H_0: \delta =1$) was rejected at the 95% confidence level (i.e., the 95% confidence interval does not include the hazard ratio $\delta =1$). For the data that were generated with $\delta =1$, this percent rejection represents the type I error. For the data generated with $\delta = 0.5$, the rejection percent provides the power.

### Type I error

There is no discernible difference in type I error between the constrained ($q=0.10$) and the highly constrained ($q=0.01$) randomizations (Table 1).

**Table 1:** Type I error rates for $g=8$ and prognostic covariates. For each analysis method, the results are provided for the unadjusted analysis ($S=0$) and the analysis adjusting for all four covariates ($S=4$)

		Cox PH Frailty		Cox PH Robust		Permutation	
ICC	Randomization^a^	*S* = 0	*S* = 4	*S* = 0	*S* = 4	*S* = 0	*S* = 4
0.05	Highly Constr.	0.006 (0.002)	0.042 (0.006)	0.006 (0.002)	0.096 (0.009)	0.011 (0.003)	0.090 (0.009)
	Constrained	0.014 (0.004)	**0.050** (0.007)	0.016 (0.004)	0.113 (0.010)	0.017 (0.004)	0.076 (0.008)
	Simple	0.076 (0.008)	**0.048** (0.007)	0.082 (0.009)	0.104 (0.010)	**0.054** (0.007)	**0.052** (0.007)
0.08	Highly Constr.	0.002 (0.001)	0.037 (0.006)	0.002 (0.001)	0.098 (0.009)	0.007 (0.003)	0.065 (0.008)
	Constrained	0.004 (0.002)	**0.045** (0.007)	0.004 (0.002)	0.113 (0.010)	0.007 (0.003)	**0.054** (0.007)
	Simple	0.078 (0.008)	**0.052** (0.007)	0.084 (0.009)	0.111 (0.010)	0.061 (0.008)	0.042 (0.006)
0.14	Highly Constr.	0.000 (0.000)	**0.051** (0.007)	0.000 (0.000)	0.110 (0.010)	0.001 (0.001)	0.030 (0.005)
	Constrained	0.000 (0.000)	**0.046** (0.007)	0.001 (0.001)	0.119 (0.010)	0.003 (0.002)	0.034 (0.006)
	Simple	0.084 (0.009)	**0.055** (0.007)	0.087 (0.009)	0.117 (0.010)	**0.056** (0.007)	**0.053** (0.007)

Monte Carlo standard errors are shown in parentheses. Results that include the nominal value of $\alpha = 0.05$ within a 95% confidence interval are in bold

^a^For randomization, “highly constrained” refers to $q=0.01$, “constrained” refers to $q=0.10$, and “simple” refers to $q=1$

**Fig. 1:**
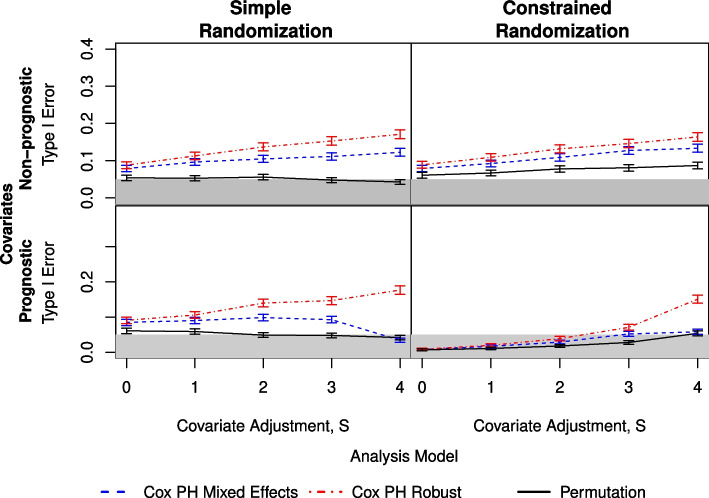
Comparison of three analysis methods as measured by type I error under simple (left) and constrained (right) randomization with prognostic covariates (lower) and non-prognostic covariates (upper). Here, there are $g=8$ clusters per arm and an ICC of 0.08. The shaded area indicates the nominal $\alpha = 0.05$

**Fig. 2:**
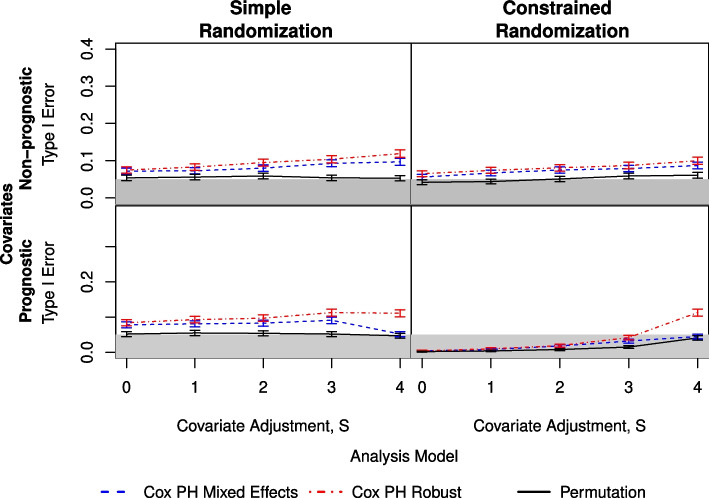
Comparison of three analysis methods as measured by type I error under simple (left) and constrained (right) randomization with prognostic covariates (lower) and non-prognostic covariates (upper). Here, there are $g=13$ clusters per arm and an ICC of 0.08. The shaded area indicates the nominal $\alpha = 0.05$

The simulations using $g=8$ groups per arm and non-prognostic covariates (Fig. 1) showed that the analyses using the permutation method generally had a type I error that was close to the nominal value of $\alpha = 0.05$, with the exception of a small amount of inflation for the constrained randomization as S increased. The analyses using the two Cox proportional hazards model-based methods consistently had an inflated type I error, with the model using a robust variance term having a higher error, particularly for simple randomization. There was little change in any method as the ICC increased. For the simulations with $g=13$ groups per arm and non-prognostic covariates (Fig. 2), the results were similar, with the overall inflation of the type I error being lower and equivalent (within the 95% confidence interval) for the two model-based methods.

For those simulations with $g=8$ and prognostic covariates (Fig. 1 and Table 1), the simple randomization results are similar to those in the non-prognostic case. An improvement in the mixed effects model is seen for low ICC values, but only when all four covariates are adjusted for in the analysis. For the constrained randomization, the permutation method and mixed effects model are both conservative across $S=\{0,1,2,3\}$. The model using a robust variance term is also conservative for $S \le 2$, but is inflated at $S=4$. The results for $g=13$ groups per arm are similar (Fig. 2), with overall inflation being lower for all analysis methods.

The figures provided here are limited to ICC = 0.08 ($\varvec{\beta } = 0.35$) and $q=0.10$. The results from ICC $=\{0.05, 0.14\}$ and $q=0.01$ are similar and are provided in the Supplemental Material in Tables S1.1-S1.3 and Figures S1.1-S1.10.

### Power

We provide the following results for the power of the tests with the cautionary note that they may not be accurate for the cases where type I error is conservative or inflated. As was the case for the type I error, the constrained randomizations ($q=0.10$) and highly constrained randomizations ($q=0.01$) provided similar results for the power. Figures for the highly constrained randomizations, as well as those for ICC $=\{0.05, 0.14\}$, can be found in the Supplemental Material (Figures S2.1-S2.11) along with tables of results for $g=8$ (Tables S2.1-S2.2) and $g=13$ (Tables S2.3-S2.4).

**Fig. 3:**
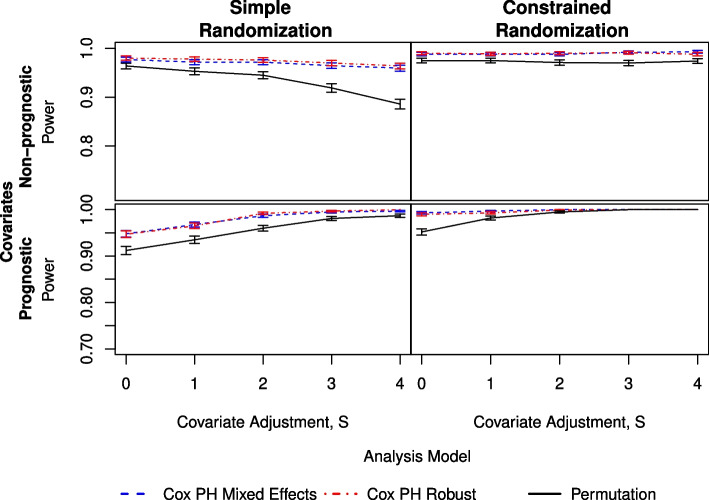
Comparison of three analysis methods as measured by power under simple (left) and constrained (right) randomization with prognostic covariates (lower) and non-prognostic covariates (upper). Here, there are $g=8$ clusters per arm and an ICC of 0.08

When $g=8$ and the covariates are non-prognostic (Fig. 3), all three analysis methods have at least 95% power for the constrained randomizations and for both ICC $=\{0.05, 0.08\}$. However, when ICC $= 0.14$ (Figure S2.4), the model-based analyses remain above 90%, and the permutation method drops down to approximately 89% across *S*. For the simple randomizations, the power from the permutation method drops considerably as *S* increases. There is also some drop in power for the two Cox model-based methods at higher ICC as *S* increases. Li et al. [8] saw a similar trend and referred to this as “covariate over-adjustment.” However, it is not immediately clear why the permutation test performs worse than the two model-based analyses in the time-to-event scenario. For $g=13$, all methods provide power greater than 95% across all values of *S* and ICC for constrained randomizations.

The simulations with $g=8$ and prognostic covariates (Fig. 3) show that the two Cox model-based analysis methods have higher power for the simple randomizations, whereas the constrained randomizations result in the three models converging as *S* increases. All methods show improvement with an increase in covariate adjustment, becoming substantial at ICC $=0.14$. The two model-based methods are consistent with each other in terms of power. For $g=13$, power is generally improved over $g=8$ (Tables S2.1-S2.4). All three methods—for all ICC values—provide power near 100% for the constrained randomizations and power above 90% for the simple randomizations.

## Motivating example

The TIRS Trial [19] (ClinicalTrials.gov identifier: NCT04343521) aims to investigate the efficacy of Targeted Indoor Residual Spraying (TIRS) for the prevention of *Aedes*-borne viruses (ABVs) in children in Mérida, Mexico. Targeted Indoor Residual Spraying is referred to as such because the insecticide used for vector control is sprayed only on indoor surfaces below 1.5m and particularly those surfaces where mosquitoes are most likely to rest (e.g., the underside of tables and chairs). This allows for deployment in less than 18% of the time and using less than 30% of the insecticide volume when compared to standard indoor residual spraying. The use of a cluster randomized trial for this study is not only logistically more feasible for enrolling the required number of children and implementing TIRS, but is also needed to reduce contamination between treatment arms since mosquitoes could travel between treated and untreated households. This is an unblinded, parallel, two-arm cluster randomized trial in which the method of employing TIRS prior to the beginning of the ABV transmission season is being compared to the standard reactive vector control methods (truck-mounted ultra-low volume spraying and larviciding) that typically occur only after an incidence of disease. The primary outcome of interest in this study is the time-to-event for laboratory confirmed dengue, Zika, or chikungunya viruses in enrolled children between the ages of two and 15 years.

A cluster in this trial is defined as an area of roughly 5x5 city blocks within a census tract. There are 25 clusters in each arm of the trial. Covariate-constrained randomization was used in the design phase [20] to ensure balance (within a 10% difference) between the two arms with respect to historical ABV transmission, population size, population density, and the percent of employed population. Additionally, the number of clusters in each of four geographic sectors of the city was balanced within $\pm 1$. For the TIRS trial, this balance was achieved by selecting a final allocation pattern from only those patterns in which–for each covariate–the covariate mean from arm A divided by the covariate mean of arm B was greater than 1/1.1 and less than 1.1. This is an alternative to using the imbalance metric described by Eq. (3). Validity was confirmed by ensuring that there were no pairs of two clusters that were assigned to the same arm in $\le 25\%$ or $\ge 75\%$ of the final balanced randomization space.

Of note, the TIRS trial has a nested clustering structure due to many households containing multiple enrolled children. In light of this, the planned analysis method for the primary outcome was a Cox proportional hazards model with a robust variance term to account for the hierarchical clustering.

**Fig. 4:**
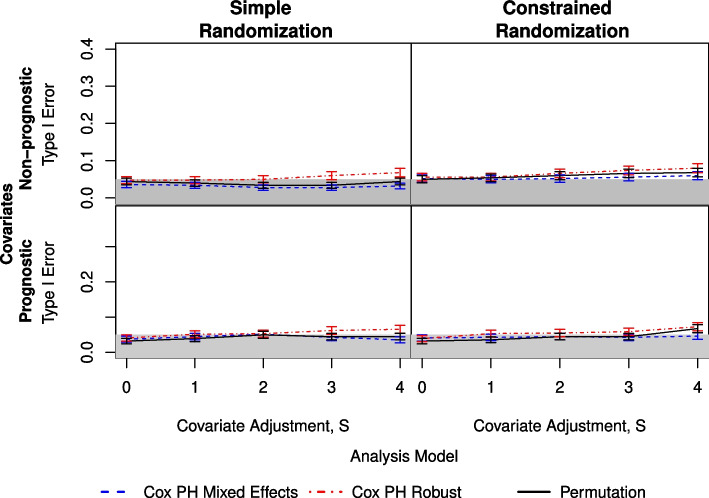
Comparison of three analysis methods as measured by type I error under simple (left) and constrained (right) randomization with prognostic covariates (lower) and non-prognostic covariates (upper). Here, there are $g=25$ clusters per arm and an ICC of 0.05, as in the TIRS Trial. The shaded area indicates the nominal $\alpha = 0.05$

**Fig. 5:**
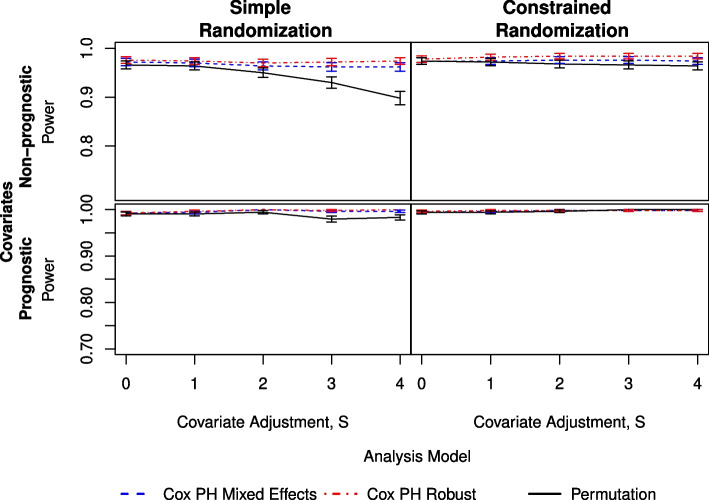
Comparison of three analysis methods as measured by power under simple (left) and constrained (right) randomization with prognostic covariates (lower) and non-prognostic covariates (upper). Here, there are $g=25$ clusters per arm and an ICC of 0.05, as in the TIRS Trial

Additional simulations were performed using the data generating mechanism described in the Methods section, this time using $g=25$ and $q=0.10$. The number of subjects per cluster was maintained at 100 to reflect the trial’s target number of 92 children per cluster. For the power calculations conducted in the design phase of the trial, the ICC value was set to 0.035 based on previous studies [19], and the expected cumulative incidence over two years was 4%. Therefore, the $n_{sim}=500$ simulations performed used that incidence and an ICC of 0.05. The results of these additional simulations are presented in Figs. 4 and 5.

Based on these results, the TIRS trial contains a sufficient number of clusters to allow for the use of any of the three analysis methods with no considerable penalty in type I error for adjustment—or overadjustment—of covariates. While there is a small penalty in power for overadjustment of covariates, the power remains above 95% for all three methods in either case. Given this, the analysis method can be driven primarily by the differentiation between a marginal and a conditional treatment effect.

## Discussion

We evaluate the performance of covariate-constrained randomization and three analysis methods for time-to-event outcomes, as indicated by measured type I error and power. While the primary goal of covariate-constrained randomization is usually the avoidance of an unlucky and unbalanced randomization scheme [4], the power advantages of covariate-constrained randomization over simple randomization are once again demonstrated by these simulations. Noting that power estimates are not reliable when type I error is not maintained, the permutation test provides the best comparison between the two randomization methods. The permutation test remains near the nominal type I error rate in all cases with the exception of being overly conservative in the unadjusted case for constrained randomization. Comparing the power of the permutation test between the two randomization methods, we see that covariate-constrained randomization maintains a higher power for both the unadjusted and adjusted cases with no penalty for adjusting for non-prognostic covariates. These results are more pronounced for the simulations with only eight groups per trial arm compared with 13.

Another primary goal of this study was a comparison of unadjusted analyses and analyses that adjust for the covariates which were balanced in the design phase. The simulation results indicate that there is inflation of type I error as the number of non-prognostic covariates included is increased, but the permutation test remains near a rejection rate of $\alpha = 0.05$ across the number of included covariates. However, all methods are overly conservative (rejecting H_0_ at a lower rate) when unadjusted in the presence of prognostic covariates. The mixed effects model and the permutation test perform well when fully adjusted, providing the nominal type I error rate. For trials with only eight groups per arm and the highest ICC, there was a considerable increase in power when adjusting for covariates, with only a small penalty for overadjustment of the model by including non-prognostic covariates. Outside of this distinction, there was little change in the results as ICC was varied.

As there was no obvious choice for a permutation test to be used with censored time-to-event data that also included adjustment for multiple covariates, a test had to be derived for these simulations. This permutation test performed well in terms of type I error, with the improvement over the two Cox model-based methods being more substantial with only eight groups per trial arm compared with 13. This test also provided power comparable to the model-based methods in most cases.

To summarize these results, a cluster randomized trial in which there are a small number of clusters enrolled can generally benefit from covariate-constrained randomization so long as validity of the final randomization is confirmed [4, 20]. Assuming that the researchers are confident that the balancing covariates are prognostic of the outcome of interest, the permutation test provided here is the best candidate for maintaining the nominal type I error—but only if the balancing covariates are adjusted for in the analysis. The adjusted analysis also provides power above 95%. If the researchers have unknowingly used non-prognostic covariates, there is only a slight inflation of type I error with the permutation test and no loss in power—as long as covariate-constrained randomization was used.

The results presented here are generally in agreement with those found by Li et al. [8] Of note, the permutation test does not suffer as much from a loss in power in the survival analysis framework. However, the permutation test matches the model-based tests in being overly conservative in the unadjusted case for constrained randomization. With respect to analysis methods, these simulations showed little change in rejection rate between the constrained randomizations ($q=0.10$) and the highly constrained randomizations ($q=0.01$).

The time-to-event data for the simulations in this study were generated using an exponential proportional hazards model with non-informative right censoring. Thus, these results do not necessarily extend to cases where the proportional hazards assumption is invalid (or is time varying) or data with other forms of censoring—particularly informative censoring. Furthermore, there was no exploration of other survival analysis frameworks such as repeated measures or multiple outcomes. Additional research is needed to explore the use of the permutation test derived here in those situations. Finally, these simulations only included adjustments for the cluster-level covariates that were used for balancing in the design phase. However, additional cluster-level covariates and individual-level covariates may also be of interest and may provide different results.

Various methods exist for ensuring balance between trial arms of predictive covariates and can be useful in reducing variation in the data and improving the power of a trial without requiring additional resources. For each of these methods though, it is important to operate within its limitations, and there may be ways to maximize its benefits. This simulation study provides new methods and evidence for ways to analyze time-to-event data from covariate-constrained randomized trials while minimizing inflation of type I error and loss of power.

## Supplementary Information

Supplementary Material 1.

## Data Availability

The datasets generated, used and/or analysed during the current study are available from the corresponding author on reasonable request. The code used to generate and analyse the data can be found at https://github.com/amycrisp/TTEsimulations.
