# Supplementary material for: Analysis methods for covariate-constrained cluster randomized trials with time-to-event outcomes
Source: BMC Med Res Methodol. 2025 Jan 22;25:16. doi: 10.1186/s12874-025-02465-w (PMC11753003; doi:10.1186/s12874-025-02465-w)
Supplement: Supplementary file 1 — Supplementary Material 1. [file 12874_2025_2465_MOESM1_ESM.pdf]

Analysis Methods for Covariate-Constrained Cluster Randomized Trials with Time-to-Event Outcomes – Supplemental Material

Amy M. Crisp[1*], M. Elizabeth Halloran[2,3], Matt D.T. Hitchings[1], Ira M. Longini[1], Natalie E. Dean[4]

[1*]Department of Biostatistics, University of Florida, Gainesville, Florida, USA.
[2]Department of Biostatistics, University of Washington, Seattle, Washington, USA.
[3]Vaccine and Infectious Diseases Division, Fred Hutchinson Cancer Center, Seattle, Washington, USA.
[4]Department of Biostatistics and Bioinformatics, Emory University, Atlanta, Georgia, USA.

*Corresponding author(s). E-mail(s): amy.crisp@jax.ufl.edu;

S1 Type I Error

S1.1 g=8

Table S1.1 Type I error rates for $g = 8$ and non-prognostic covariates. For each analysis method, the results are provided for the unadjusted analysis ($S = 0$) and the analysis adjusting for all four covariates ($S = 4$). Monte Carlo standard errors are shown in parentheses. Results that include the nominal value of $\alpha = 0.05$ within a 95% confidence interval are in bold.

		Cox PH Frailty		Cox PH Robust		Permutation	
ICC	Randomization[a]	$S = 0$	$S = 4$	$S = 0$	$S = 4$	$S = 0$	$S = 4$
0.05	Highly Constr.	0.076 (0.008)	0.104 (0.010)	0.095 (0.009)	0.158 (0.012)	0.064 (0.008)	0.100 (0.009)
	Constrained	0.071 (0.008)	0.119 (0.010)	0.085 (0.009)	0.158 (0.012)	**0.051** (0.007)	0.089 (0.009)
	Simple	0.072 (0.008)	0.122 (0.010)	0.092 (0.009)	0.186 (0.012)	**0.049** (0.007)	**0.049** (0.007)
0.08	Highly Constr.	0.083 (0.009)	0.126 (0.010)	0.096 (0.009)	0.167 (0.012)	0.059 (0.007)	0.100 (0.009)
	Constrained	0.079 (0.009)	0.134 (0.011)	0.089 (0.009)	0.164 (0.012)	0.061 (0.008)	0.087 (0.009)
	Simple	0.079 (0.009)	0.122 (0.010)	0.088 (0.009)	0.171 (0.012)	**0.054** (0.007)	0.043 (0.006)
0.14	Highly Constr.	0.082 (0.009)	0.153 (0.011)	0.095 (0.009)	0.178 (0.012)	0.061 (0.008)	0.108 (0.010)
	Constrained	0.080 (0.009)	0.128 (0.011)	0.089 (0.009)	0.140 (0.011)	**0.057** (0.007)	0.087 (0.009)
	Simple	0.067 (0.008)	0.123 (0.010)	0.081 (0.009)	0.177 (0.012)	0.040 (0.006)	**0.046** (0.007)

[a]For randomization, “highly constrained” refers to $q = 0.01$, “constrained” refers to $q = 0.10$, and “simple” refers to $q = 1$.

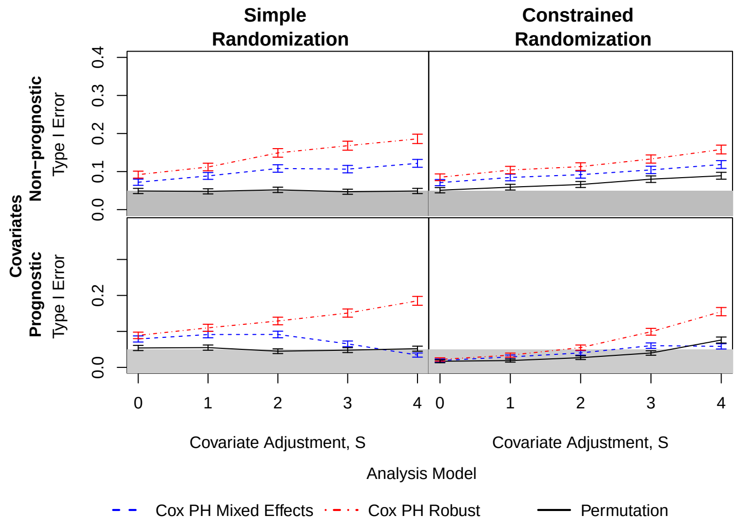

Fig. S1.1 Comparison of three analysis methods as measured by type I error under simple (left) and constrained (right) randomization with prognostic covariates (lower) and non-prognostic covariates (upper). Here, there are $g = 8$ clusters per arm and an ICC of 0.05. The shaded area indicates the nominal $\alpha = 0.05$.

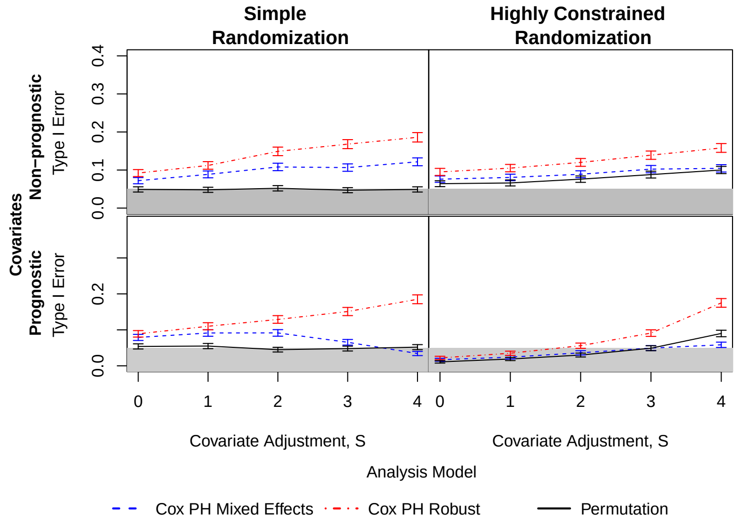

Fig. S1.2 Comparison of three analysis methods as measured by type I error under simple (left) and highly constrained (right) randomization with prognostic covariates (lower) and non-prognostic covariates (upper). Here, there are $g = 8$ clusters per arm and an ICC of 0.05. The shaded area indicates the nominal $\alpha = 0.05$.

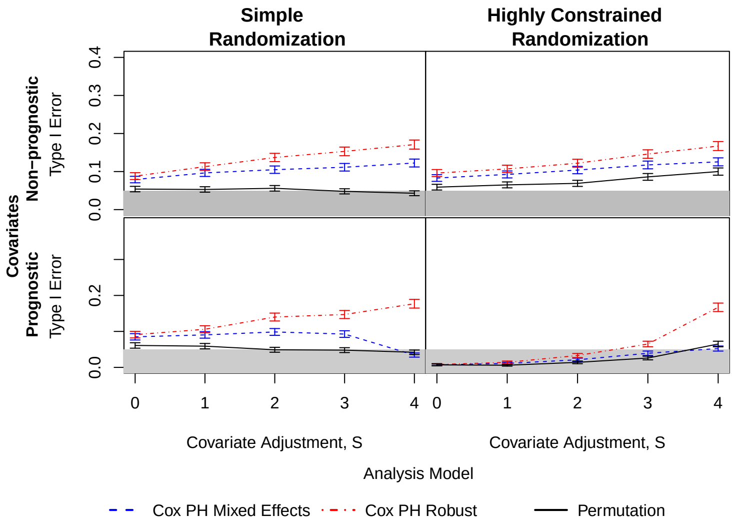

Fig. S1.3 Comparison of three analysis methods as measured by type I error under simple (left) and highly constrained (right) randomization with prognostic covariates (lower) and non-prognostic covariates (upper). Here, there are $g = 8$ clusters per arm and an ICC of 0.08. The shaded area indicates the nominal $\alpha = 0.05$.

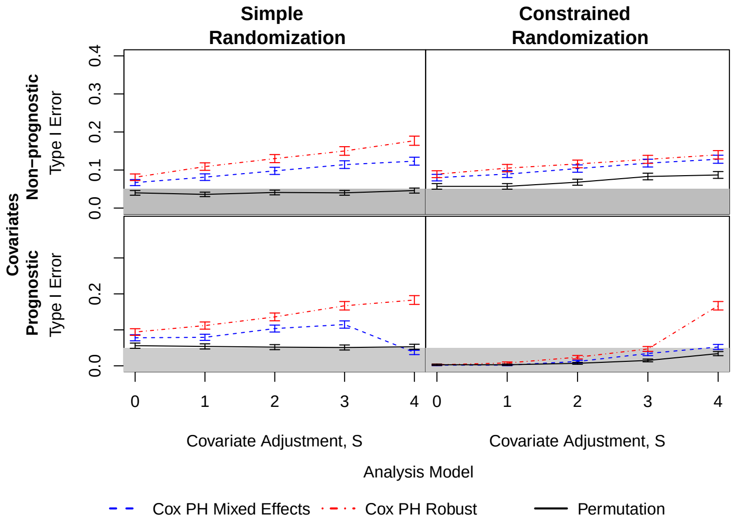

Fig. S1.4 Comparison of three analysis methods as measured by type I error under simple (left) and constrained (right) randomization with prognostic covariates (lower) and non-prognostic covariates (upper). Here, there are $g = 8$ clusters per arm and an ICC of 0.14. The shaded area indicates the nominal $\alpha = 0.05$.

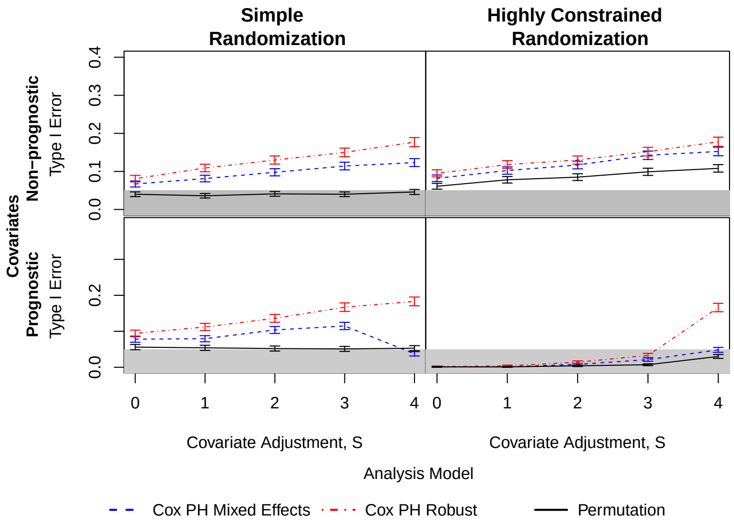

Fig. S1.5 Comparison of three analysis methods as measured by type I error under simple (left) and highly constrained (right) randomization with prognostic covariates (lower) and non-prognostic covariates (upper). Here, there are $g = 8$ clusters per arm and an ICC of 0.14. The shaded area indicates the nominal $\alpha = 0.05$.

S1.2 g=13

Table S1.2 Type I error rates for $g = 13$ and prognostic covariates. For each analysis method, the results are provided for the unadjusted analysis ($S = 0$) and the analysis adjusting for all four covariates ($S = 4$). Monte Carlo standard errors are shown in parentheses. Results that include the nominal value of $\alpha = 0.05$ within a 95% confidence interval are in bold.

		Cox PH Frailty		Cox PH Robust		Permutation	
ICC	Randomization[a]	$S = 0$	$S = 4$	$S = 0$	$S = 4$	$S = 0$	$S = 4$
0.05	Highly Constr.	0.006 (0.002)	0.042 (0.006)	0.006 (0.002)	0.096 (0.009)	0.004 (0.002)	**0.047** (0.007)
	Constrained	0.014 (0.004)	**0.050** (0.007)	0.016 (0.004)	0.113 (0.010)	0.007 (0.003)	0.064 (0.008)
	Simple	0.076 (0.008)	**0.048** (0.007)	0.082 (0.009)	0.104 (0.010)	**0.052** (0.007)	**0.046** (0.007)
0.08	Highly Constr.	0.002 (0.001)	0.037 (0.006)	0.002 (0.001)	0.098 (0.009)	0.002 (0.001)	0.036 (0.006)
	Constrained	0.004 (0.002)	**0.045** (0.007)	0.004 (0.002)	0.113 (0.010)	0.002 (0.001)	0.041 (0.006)
	Simple	0.078 (0.008)	**0.052** (0.007)	0.084 (0.009)	0.111 (0.010)	**0.052** (0.007)	**0.047** (0.007)
0.14	Highly Constr.	0.000 (0.000)	0.151 (0.007)	0.000 (0.000)	0.110 (0.010)	0.000 (0.000)	0.018 (0.004)
	Constrained	0.000 (0.000)	**0.051** (0.007)	0.000 (0.001)	0.119 (0.010)	0.001 (0.001)	0.031 (0.005)
	Simple	0.084 (0.009)	**0.055** (0.007)	0.087 (0.009)	0.117 (0.010)	0.065 (0.008)	**0.048** (0.007)

[a]For randomization, "highly constrained" refers to $q = 0.01$, "constrained" refers to $q = 0.10$, and "simple" refers to $q = 1$.

Table S1.3 Type I error rates for $g = 13$ and non-prognostic covariates. For each analysis method, the results are provided for the unadjusted analysis ($S = 0$) and the analysis adjusting for all four covariates ($S = 4$). Monte Carlo standard errors are shown in parentheses. Results that include the nominal value of $\alpha = 0.05$ within a 95% confidence interval are in bold.

		Cox PH Frailty		Cox PH Robust		Permutation	
ICC	Randomization[a]	$S = 0$	$S = 4$	$S = 0$	$S = 4$	$S = 0$	$S = 4$
0.05	Highly Constr.	0.064 (0.008)	0.086 (0.009)	0.073 (0.008)	0.102 (0.010)	**0.048** (0.007)	0.065 (0.008)
	Constrained	**0.057** (0.007)	0.075 (0.008)	0.075 (0.008)	0.087 (0.009)	**0.050** (0.007)	0.062 (0.008)
	Simple	0.061 (0.008)	0.083 (0.009)	0.070 (0.008)	0.097 (0.009)	**0.049** (0.007)	**0.048** (0.007)
0.08	Highly Constr.	0.080 (0.009)	0.108 (0.010)	0.085 (0.009)	0.117 (0.010)	0.058 (0.007)	0.085 (0.009)
	Constrained	**0.056** (0.007)	0.087 (0.009)	0.065 (0.008)	0.100 (0.009)	0.042 (0.006)	0.061 (0.008)
	Simple	0.072 (0.008)	0.097 (0.009)	0.075 (0.008)	0.119 (0.010)	**0.054** (0.007)	**0.053** (0.007)
0.14	Highly Constr.	0.072 (0.008)	0.111 (0.010)	0.080 (0.009)	0.123 (0.010)	0.058 (0.007)	0.085 (0.009)
	Constrained	0.065 (0.008)	0.093 (0.009)	0.074 (0.008)	0.106 (0.010)	**0.053** (0.007)	0.063 (0.008)
	Simple	0.068 (0.008)	0.094 (0.009)	0.078 (0.008)	0.110 (0.010)	**0.057** (0.007)	**0.054** (0.007)

[a]For randomization, "highly constrained" refers to $q = 0.01$, "constrained" refers to $q = 0.10$, and "simple" refers to $q = 1$.

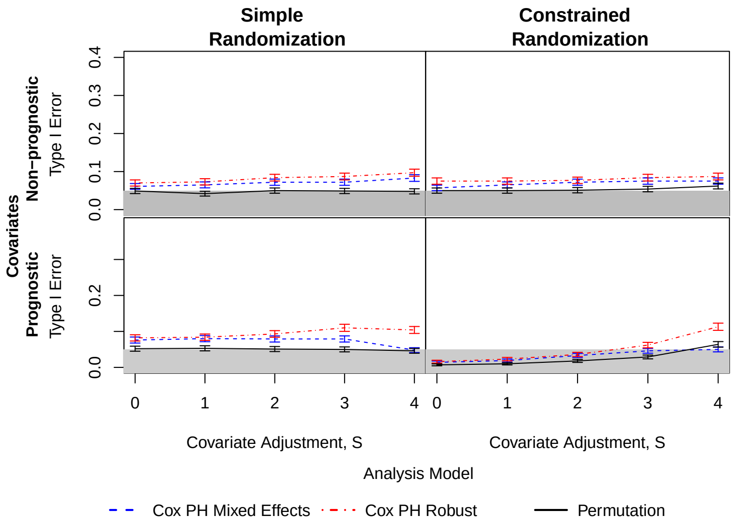

Fig. S1.6 Comparison of three analysis methods as measured by type I error under simple (left) and constrained (right) randomization with prognostic covariates (lower) and non-prognostic covariates (upper). Here, there are $g = 13$ clusters per arm and an ICC of 0.05. The shaded area indicates the nominal $\alpha = 0.05$.

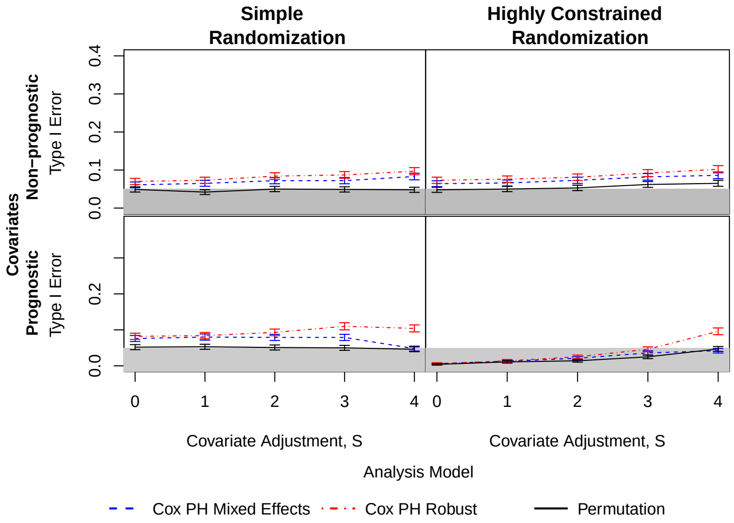

Fig. S1.7 Comparison of three analysis methods as measured by type I error under simple (left) and highly constrained (right) randomization with prognostic covariates (lower) and non-prognostic covariates (upper). Here, there are $g = 13$ clusters per arm and an ICC of 0.05. The shaded area indicates the nominal $\alpha = 0.05$.

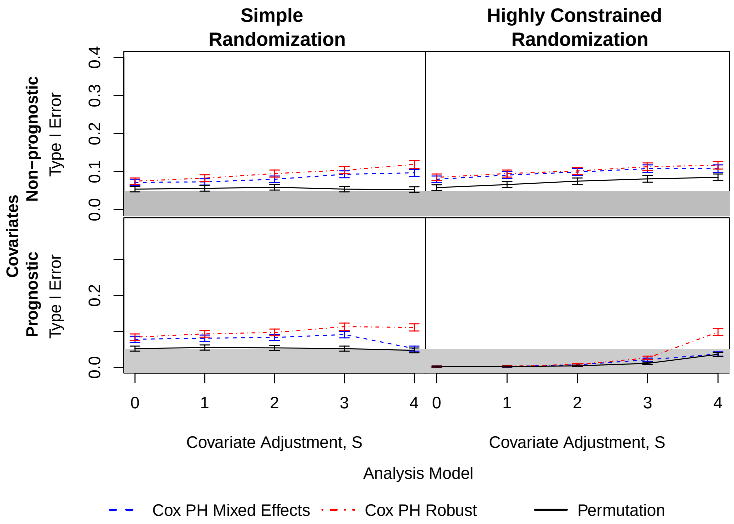

Fig. S1.8 Comparison of three analysis methods as measured by type I error under simple (left) and highly constrained (right) randomization with prognostic covariates (lower) and non-prognostic covariates (upper). Here, there are $g = 13$ clusters per arm and an ICC of 0.08. The shaded area indicates the nominal $\alpha = 0.05$.

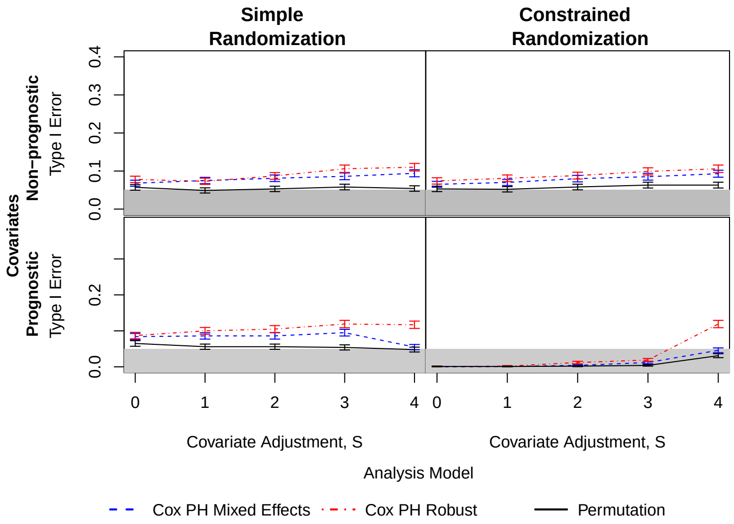

Fig. S1.9 Comparison of three analysis methods as measured by type I error under simple (left) and constrained (right) randomization with prognostic covariates (lower) and non-prognostic covariates (upper). Here, there are $g = 13$ clusters per arm and an ICC of 0.14. The shaded area indicates the nominal $\alpha = 0.05$.

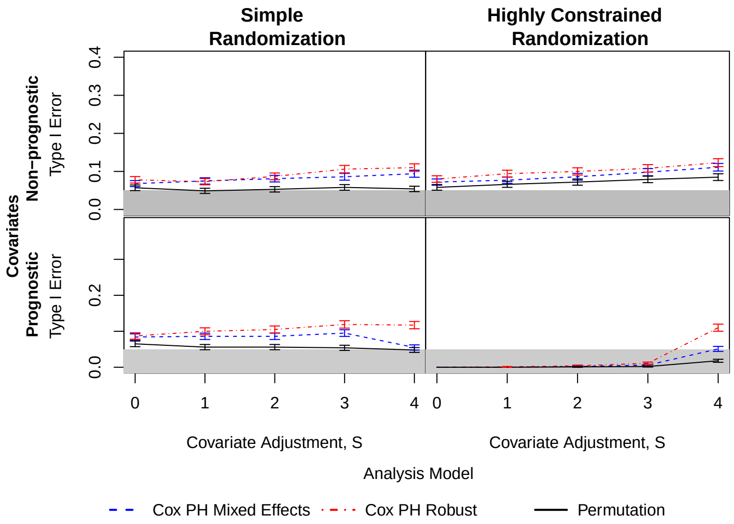

Fig. S1.10 Comparison of three analysis methods as measured by type I error under simple (left) and highly constrained (right) randomization with prognostic covariates (lower) and non-prognostic covariates (upper). Here, there are $g = 13$ clusters per arm and an ICC of 0.14. The shaded area indicates the nominal $\alpha = 0.05$.

S2 Power

S2.1 g=8

Table S2.1 Power for $g = 8$ and prognostic covariates. For each analysis method, the results are provided for the unadjusted analysis ($S = 0$) and the analysis adjusting for all four covariates ($S = 4$). Monte Carlo standard errors are shown in parentheses. Results for which the respective type I error was equal to the nominal value of $\alpha = 0.05$ within a 95% confidence interval are in bold.

		Cox PH Frailty		Cox PH Robust		Permutation	
ICC	Randomization[a]	$S = 0$	$S = 4$	$S = 0$	$S = 4$	$S = 0$	$S = 4$
0.05	Highly Constr.	1.000 (0.000)	1.000 (0.000)	1.000 (0.000)	1.000 (0.000)	0.997 (0.002)	1.000 (0.000)
	Constrained	1.000 (0.000)	**1.000** (0.000)	1.000 (0.000)	1.000 (0.000)	0.994 (0.002)	1.000 (0.000)
	Simple	0.992 (0.003)	**0.997** (0.002)	0.993 (0.003)	1.000 (0.000)	**0.983** (0.004)	**0.988** (0.003)
0.08	Highly Constr.	0.996 (0.002)	1.000 (0.000)	0.991 (0.003)	1.000 (0.000)	0.966 (0.006)	1.000 (0.000)
	Constrained	0.993 (0.003)	**1.000** (0.000)	0.990 (0.003)	1.000 (0.000)	0.952 (0.007)	**1.000** (0.000)
	Simple	0.948 (0.007)	**0.997** (0.002)	0.947 (0.007)	1.000 (0.000)	0.912 (0.009)	0.987 (0.004)
0.14	Highly Constr.	0.908 (0.009)	**1.000** (0.000)	0.885 (0.010)	1.000 (0.000)	0.752 (0.014)	1.000 (0.000)
	Constrained	0.885 (0.010)	**1.000** (0.000)	0.877 (0.010)	1.000 (0.000)	0.765 (0.013)	1.000 (0.000)
	Simple	0.787 (0.013)	**0.997** (0.002)	0.789 (0.013)	1.000 (0.000)	**0.693** (0.015)	**0.972** (0.005)

[a]For randomization, "highly constrained" refers to $q = 0.01$, "constrained" refers to $q = 0.10$, and "simple" refers to $q = 1$.

Table S2.2 Power for $g = 8$ and non-prognostic covariates. For each analysis method, the results are provided for the unadjusted analysis ($S = 0$) and the analysis adjusting for all four covariates ($S = 4$). Monte Carlo standard errors are shown in parentheses. Results for which the respective type I error was equal to the nominal value of $\alpha = 0.05$ within a 95% confidence interval are in bold.

		Cox PH Frailty		Cox PH Robust		Permutation	
ICC	Randomization[a]	$S = 0$	$S = 4$	$S = 0$	$S = 4$	$S = 0$	$S = 4$
0.05	Highly Constr.	0.993 (0.003)	0.996 (0.002)	0.995 (0.002)	0.998 (0.001)	0.979 (0.005)	0.986 (0.004)
	Constrained	0.999 (0.001)	1.000 (0.000)	0.999 (0.001)	0.999 (0.001)	**0.996** (0.002)	0.995 (0.002)
	Simple	0.993 (0.003)	0.968 (0.006)	0.995 (0.002)	0.976 (0.005)	**0.986** (0.004)	**0.938** (0.008)
0.08	Highly Constr.	0.981 (0.004)	0.984 (0.004)	0.985 (0.004)	0.989 (0.003)	0.965 (0.006)	0.969 (0.005)
	Constrained	0.988 (0.003)	0.993 (0.003)	0.990 (0.003)	0.988 (0.003)	0.975 (0.005)	0.974 (0.005)
	Simple	0.977 (0.005)	0.960 (0.006)	0.980 (0.004)	0.964 (0.006)	**0.964** (0.006)	0.886 (0.010)
0.14	Highly Constr.	0.924 (0.008)	0.937 (0.008)	0.937 (0.008)	0.948 (0.007)	0.891 (0.010)	0.914 (0.009)
	Constrained	0.933 (0.008)	0.952 (0.007)	0.942 (0.007)	0.953 (0.007)	**0.889** (0.010)	0.899 (0.010)
	Simple	0.922 (0.008)	0.883 (0.010)	0.939 (0.008)	0.906 (0.009)	0.891 (0.010)	**0.748** (0.014)

[a] For randomization, "highly constrained" refers to $q = 0.01$, "constrained" refers to $q = 0.10$, and "simple" refers to $q = 1$.

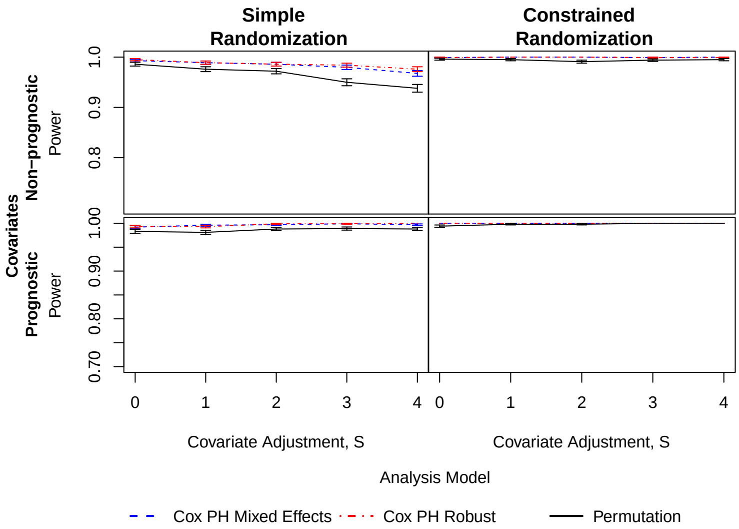

Fig. S2.1 Comparison of three analysis methods as measured by power under simple (left) and constrained (right) randomization with prognostic covariates (lower) and non-prognostic covariates (upper). Here, there are $g = 8$ clusters per arm and an ICC of 0.05.

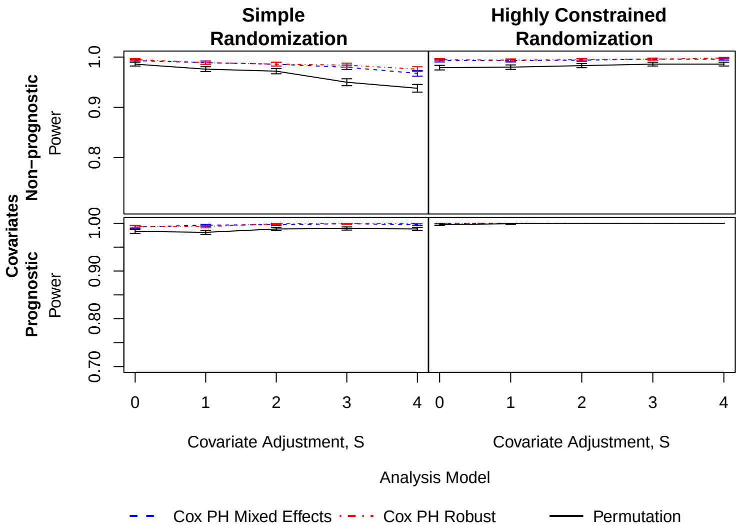

Fig. S2.2 Comparison of three analysis methods as measured by power under simple (left) and highly constrained (right) randomization with prognostic covariates (lower) and non-prognostic covariates (upper). Here, there are $g = 8$ clusters per arm and an ICC of 0.05.

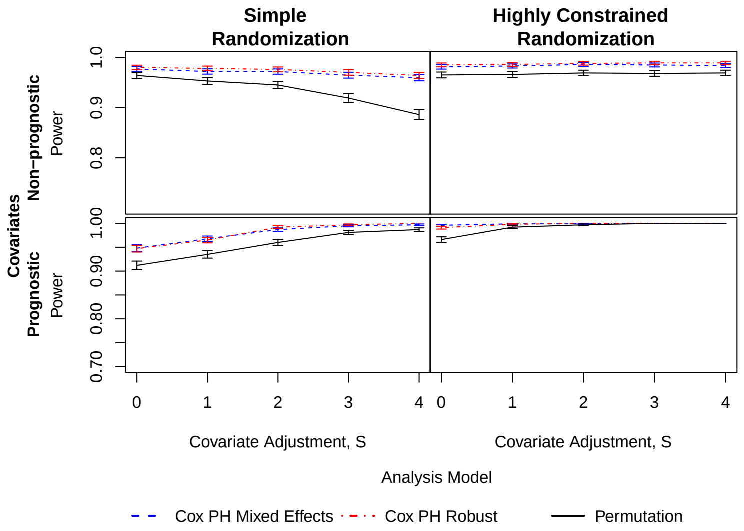

Fig. S2.3 Comparison of three analysis methods as measured by power under simple (left) and highly constrained (right) randomization with prognostic covariates (lower) and non-prognostic covariates (upper). Here, there are $g = 8$ clusters per arm and an ICC of 0.08.

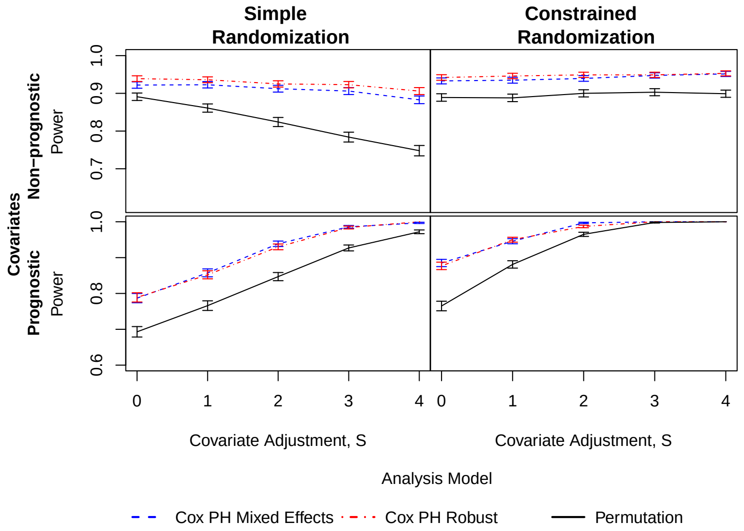

Fig. S2.4 Comparison of three analysis methods as measured by power under simple (left) and constrained (right) randomization with prognostic covariates (lower) and non-prognostic covariates (upper). Here, there are $g = 8$ clusters per arm and an ICC of 0.14. Note the change in the y-axis scale in order to accommodate the lower values for this combination.

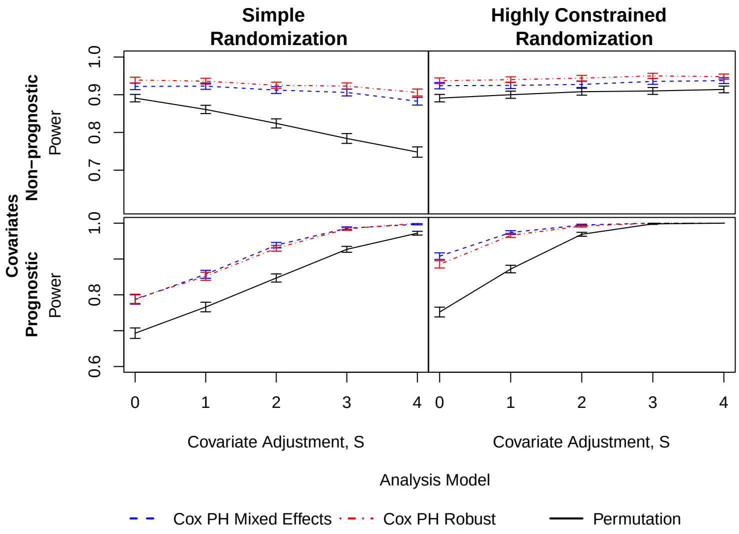

Fig. S2.5 Comparison of three analysis methods as measured by power under simple (left) and highly constrained (right) randomization with prognostic covariates (lower) and non-prognostic covariates (upper). Here, there are $g = 8$ clusters per arm and an ICC of 0.14. Note the change in the y-axis scale in order to accommodate the lower values for this combination.

S2.2 g=13

Table S2.3 Power for $g = 13$ and prognostic covariates. For each analysis method, the results are provided for the unadjusted analysis ($S = 0$) and the analysis adjusting for all four covariates ($S = 4$). Monte Carlo standard errors are shown in parentheses. Results for which the respective type I error was equal to the nominal value of $\alpha = 0.05$ within a 95% confidence interval are in bold.

		Cox PH Frailty		Cox PH Robust		Permutation	
ICC	Randomization[a]	$S = 0$	$S = 4$	$S = 0$	$S = 4$	$S = 0$	$S = 4$
0.05	Highly Constr.	1.000 (0.000)	1.000 (0.000)	1.000 (0.000)	1.000 (0.000)	1.000 (0.000)	**1.000** (0.000)
	Constrained	1.000 (0.000)	**1.000** (0.000)	1.000 (0.000)	1.000 (0.000)	1.000 (0.000)	1.000 (0.000)
	Simple	1.000 (0.000)	**1.000** (0.000)	1.000 (0.000)	1.000 (0.000)	**1.000** (0.000)	**1.000** (0.000)
0.08	Highly Constr.	1.000 (0.000)	1.000 (0.000)	1.000 (0.000)	1.000 (0.000)	1.000 (0.000)	1.000 (0.000)
	Constrained	1.000 (0.000)	**1.000** (0.000)	1.000 (0.000)	1.000 (0.000)	1.000 (0.000)	1.000 (0.000)
	Simple	0.996 (0.002)	**1.000** (0.000)	0.994 (0.002)	1.000 (0.000)	**0.989** (0.003)	**1.000** (0.000)
0.14	Highly Constr.	0.997 (0.002)	1.000 (0.000)	0.996 (0.002)	1.000 (0.000)	0.989 (0.003)	1.000 (0.000)
	Constrained	0.992 (0.003)	**1.000** (0.000)	0.989 (0.003)	1.000 (0.000)	0.973 (0.005)	1.000 (0.000)
	Simple	0.936 (0.008)	**1.000** (0.000)	0.926 (0.008)	1.000 (0.000)	0.900 (0.009)	**1.000** (0.000)

[a]For randomization, "highly constrained" refers to $q = 0.01$, "constrained" refers to $q = 0.10$, and "simple" refers to $q = 1$.

Table S2.4 Power for $g = 13$ and non-prognostic covariates. For each analysis method, the results are provided for the unadjusted analysis ($S = 0$) and the analysis adjusting for all four covariates ($S = 4$). Monte Carlo standard errors are shown in parentheses. Results for which the respective type I error was equal to the nominal value of $\alpha = 0.05$ within a 95% confidence interval are in bold.

		Cox PH Frailty		Cox PH Robust		Permutation	
ICC	Randomization[a]	$S = 0$	$S = 4$	$S = 0$	$S = 4$	$S = 0$	$S = 4$
0.05	Highly Constr.	1.000 (0.000)	1.000 (0.000)	1.000 (0.000)	1.000 (0.000)	**1.000** (0.000)	1.000 (0.000)
	Constrained	**1.000** (0.000)	1.000 (0.000)	1.000 (0.000)	0.999 (0.001)	**1.000** (0.000)	0.999 (0.001)
	Simple	1.000 (0.000)	1.000 (0.000)	1.000 (0.000)	1.000 (0.000)	**1.000** (0.000)	**1.000** (0.000)
0.08	Highly Constr.	1.000 (0.000)	1.000 (0.000)	1.000 (0.000)	1.000 (0.000)	1.000 (0.000)	1.000 (0.000)
	Constrained	**1.000** (0.000)	0.999 (0.001)	0.999 (0.001)	0.999 (0.001)	0.999 (0.001)	0.999 (0.001)
	Simple	1.000 (0.000)	0.998 (0.001)	1.000 (0.000)	0.997 (0.002)	**1.000** (0.000)	**0.994** (0.002)
0.14	Highly Constr.	0.993 (0.003)	0.998 (0.001)	0.996 (0.002)	0.998 (0.001)	0.990 (0.003)	0.994 (0.002)
	Constrained	0.991 (0.003)	0.996 (0.002)	0.996 (0.002)	0.996 (0.002)	**0.985** (0.004)	0.992 (0.003)
	Simple	0.987 (0.004)	0.971 (0.005)	0.989 (0.003)	0.972 (0.005)	**0.977** (0.005)	**0.958** (0.006)

[a]For randomization, "highly constrained" refers to $q = 0.01$, "constrained" refers to $q = 0.10$, and "simple" refers to $q = 1$.

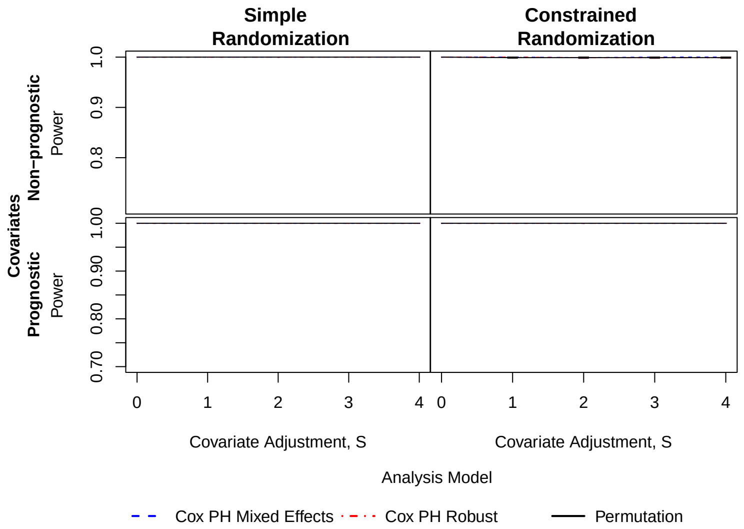

Fig. S2.6 Comparison of three analysis methods as measured by power under simple (left) and constrained (right) randomization with prognostic covariates (lower) and non-prognostic covariates (upper). Here, there are $g = 13$ clusters per arm and an ICC of 0.05.

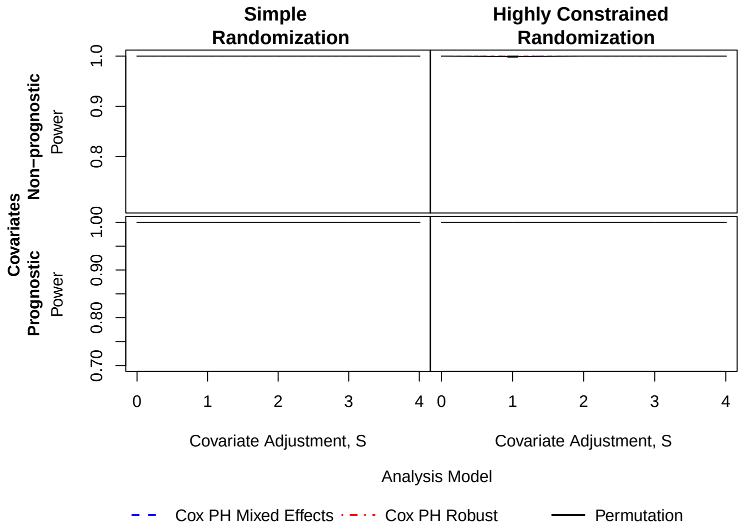

Fig. S2.7 Comparison of three analysis methods as measured by power under simple (left) and highly constrained (right) randomization with prognostic covariates (lower) and non-prognostic covariates (upper). Here, there are $g = 13$ clusters per arm and an ICC of 0.05.

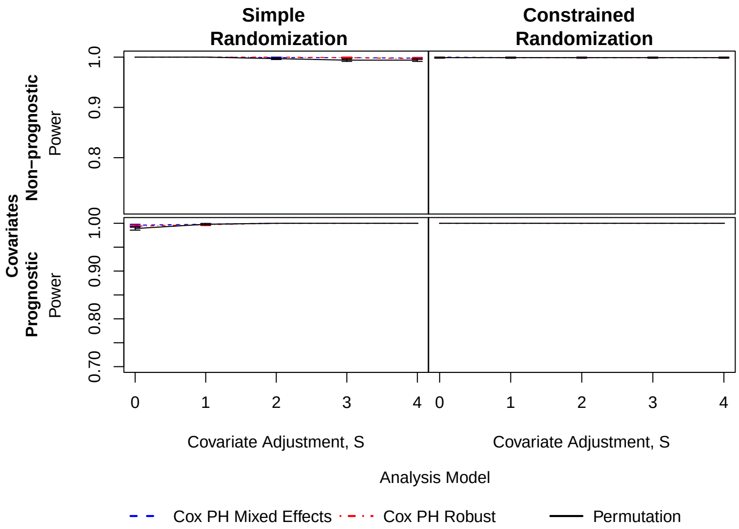

Fig. S2.8 Comparison of three analysis methods as measured by power under simple (left) and constrained (right) randomization with prognostic covariates (lower) and non-prognostic covariates (upper). Here, there are $g = 13$ clusters per arm and an ICC of 0.08.

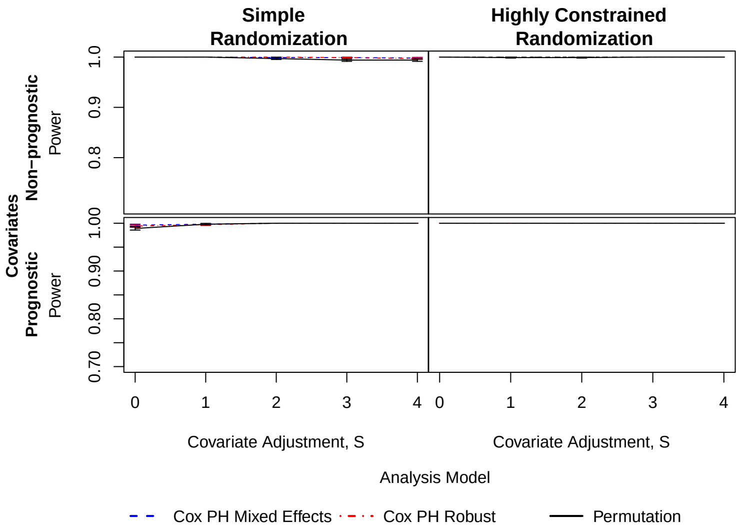

Fig. S2.9 Comparison of three analysis methods as measured by power under simple (left) and highly constrained (right) randomization with prognostic covariates (lower) and non-prognostic covariates (upper). Here, there are $g = 13$ clusters per arm and an ICC of 0.08.

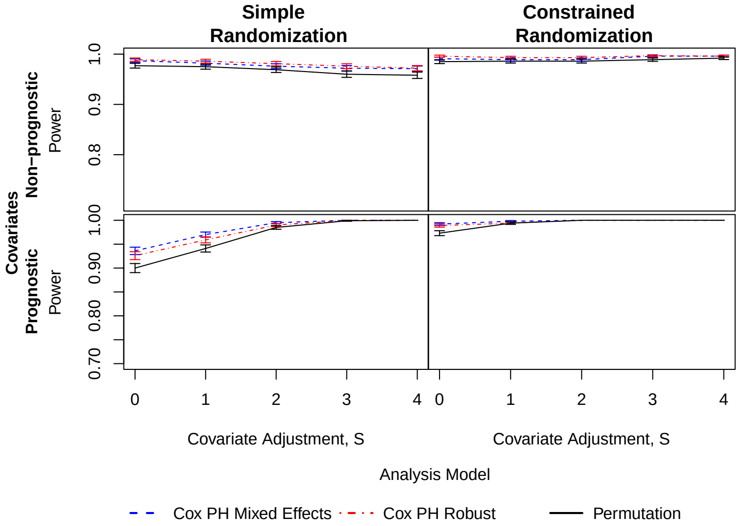

Fig. S2.10 Comparison of three analysis methods as measured by power under simple (left) and constrained (right) randomization with prognostic covariates (lower) and non-prognostic covariates (upper). Here, there are $g = 13$ clusters per arm and an ICC of 0.14.

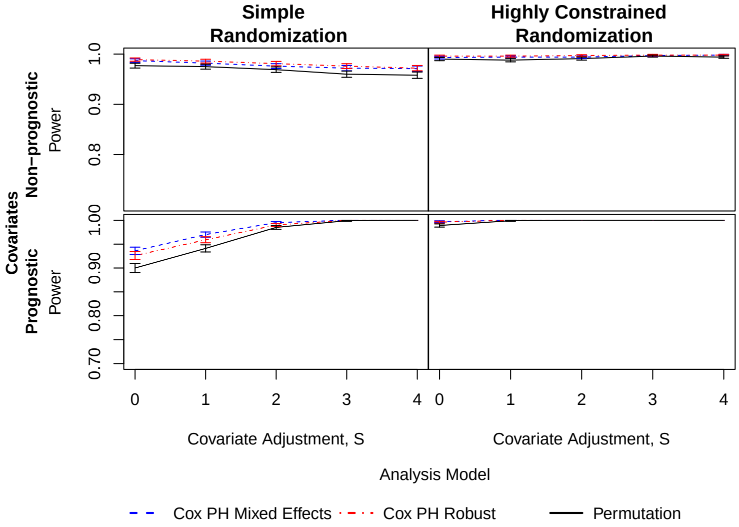

Fig. S2.11 Comparison of three analysis methods as measured by power under simple (left) and highly constrained (right) randomization with prognostic covariates (lower) and non-prognostic covariates (upper). Here, there are $g = 13$ clusters per arm and an ICC of 0.14.
